# Does the SUVmax of FDG-PET/CT Correlate with the ADC Values of DWI in Musculoskeletal Malignancies?

**DOI:** 10.5334/jbsr.2378

**Published:** 2021-02-22

**Authors:** Mesut Ozturk, Ahmet Veysel Polat, Fevziye Canbaz Tosun, Mustafa Bekir Selcuk

**Affiliations:** 1 Samsun Gazi State Hospital, TR; 2 Department of Radiology, Ondokuz Mayis University Faculty of Medicine, TR; 3 Department of Nuclear Medicine, Ondokuz Mayis University Faculty of Medicine, TR

**Keywords:** PET-CT, diffusion-weighted MRI, bone neoplasm, soft tissue sarcoma, correlation study

## Abstract

**Purpose:**

To evaluate the correlation of maximum standardized uptake values (SUV_max_) of ^18^F-Fluorodeoxyglucose-positron emission tomography/computed tomography (FDG-PET/CT) with the apparent diffusion coefficient (ADC) of diffusion weighted imaging (DWI) in musculoskeletal malignancies.

**Methods:**

Institutional ethics committee approved this retrospective study. Twenty-seven patients (mean age: 44.85 ± 24.07; 17 men and 10 women) with a total of 29 musculoskeletal tumors underwent both FDG-PET/CT and DWI between January 2017 and March 2020. Region-of-interest (ROI)-based maximal standardized uptake values (SUV_max_) of the tumors were measured on FDG-PET/CT images. Two radiologists measured lesions’ mean and minimum apparent diffusion coefficient (ADC_mean_ and ADC_min_) using five distinct ROIs on DWI images. Pearson correlation analysis was used to assess the correlation between SUV_max_ and ADC values.

**Results:**

There were 18 soft tissue tumors (62.1%) and 11 bone tumors (37.9%) with a mean maximum diameter of 9.4 ± 6.2 cm. The mean SUV_max_, ADC_mean_ and ADC_min_ of the whole lesions were 12.93 ± 9.63, 0.85 ± 0.28 × 10^–3^mm^2^/s and 0.61 ± 0.27 × 10^–3^mm^2^/s, respectively. SUV_max_ had a weak correlation with tumor maximum diameter (r = 0.378, p = 0.043), whereas ADC_mean_ and ADC_min_ had none. There was strong inverse correlation between SUV_max_ and both ADC_mean_ (r = –0.616, p < 0.001) and ADC_min_ (r = –0.638, p < 0.001).

**Conclusion:**

In musculoskeletal tumors, quantitative markers of FDG uptake and diffusion restriction strongly correlate.

## Introduction

Diffusion-weighted imaging (DWI) and ^18^F-Fluorodeoxy glucose-positron emission tomography/computed tomography (FDG-PET/CT) are commonly used imaging modalities for the evaluation of various oncologic processes, including musculoskeletal tumors [1,2,3,4,5,6]. Many studies in the literature have reported that both imaging modalities were capable of differentiating benign from malignant tumors. FDG uptake, expressed as maximum standardized uptake value (SUV_max_), increases in malignant tumor cells, and has been reported to correlate with aggressiveness [7,8,9,10,11]. On the other hand, apparent diffusion coefficient (ADC) derived from DWI has been also reported to have a role in predicting malignancy and aggressiveness of the lesion [12,13,14].

Previous studies in the literature reported significant correlation between histopathological findings and SUV_max_ of the tumors [9,10,15,16]. FDG-PET/CT reflects the glycolytic activity in a tumor region, thus most cellular activities including mitosis. As DWI shows the diffusivity of water molecule in the examined tissue, restricted diffusion also reflects cellular abundance and decreased extracellular space. As both DWI and FDG-PET/CT are functional imaging techniques that are used to evaluate tumor characteristics, one may hypothesize that these two imaging modalities may show a significant correlation. There are several studies in the literature investigating the correlation of these two modalities in different organ malignancies, but few studies include musculoskeletal malignancies [7,9,17,18]. The aim of this study was to evaluate whether there is a correlation between the SUV_max_ of FDG-PET/CT and ADC values of DWI in musculoskeletal tumors.

## Methods

The institutional ethics committee approval was obtained for this retrospective study (OMUKAEK2020/16) and the requirement for informed consent was waived. The standards for the reporting of diagnostic accuracy studies (STARD) were used [19].

### Patient Selection

**Figure 1:**
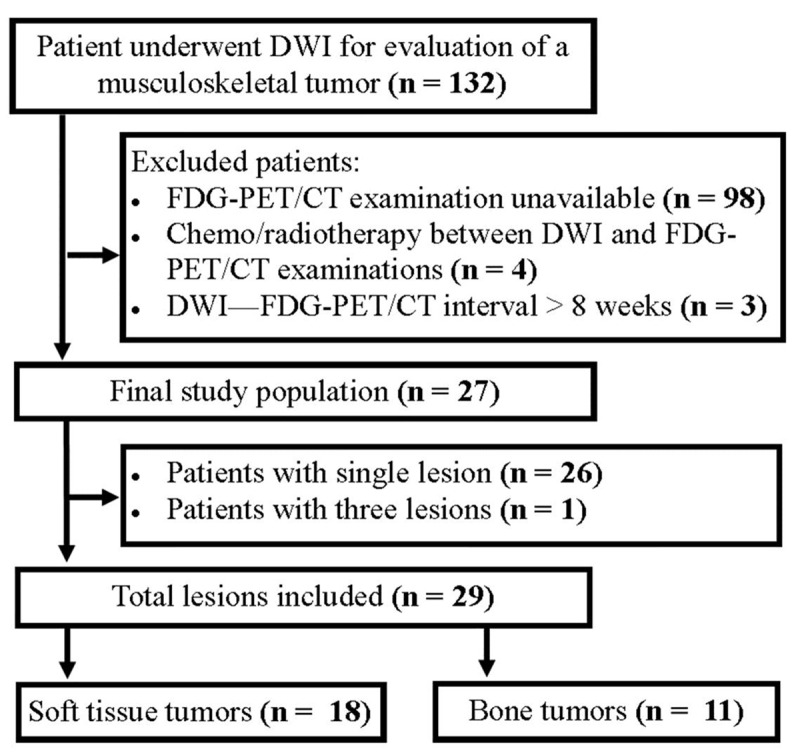
Flowchart of the study.

A retrospective search of our hospital database was performed to identify patients who underwent magnetic resonance imaging (MRI) including DWI for the evaluation of a musculoskeletal tumor between January 2017 and March 2020, and 132 patients were found. Among those, 98 were excluded as they had no FDG-PET/CT examinations available; 4 were excluded as they underwent chemo/radiotherapy within the interval period between DWI and FDG-PET/CT examinations; 3 patients were excluded as the DWI and FDG-PET/CT examination interval period was more than eight weeks. As a result, 27 patients (17 men and 10 women) with a mean age of 44.85 ± 24.07 (range: 2–82) met the enrollment criteria. Twenty-six patients had a single tumor whereas one patient had three tumors. Therefore, 29 musculoskeletal tumors were involved in the study (***Figure 1***). The mean interval period between DWI and FDG-PET/CT was 18 ± 12 days (range: 1–45).

### MRI and FDG-PET/CT Acquisitions

MRI examinations of the patients were performed with a 3T system (Ingenia, Philips, Netherlands). Different coils and imaging protocols were used according to the body site involved. The standard protocol for a musculoskeletal malignancy in the authors’ center included a longitudinal fat-suppressed T2-weighted (T2W) TSE sequence, axial T1W and T2W TSE sequences, longitudinal and axial fat-suppressed contrast-enhanced T1W TSE sequences. The DWI sequence was obtained before the contrast-enhanced sequences with the following parameters: TR/TE, 1500/80 ms; matrix, 116 × 114; field of view, 350 × 350 mm; slice thickness, 5 mm; gap, 1 mm; sensitivity encoding factor, 2; b values, 0, 200, 400 and 800s/mm^2^. ADC maps were calculated from images with b values of 0 and 800 s/mm^2^.

FDG-PET/CT scans from the top of the head through the feet of the patients were performed using a hybrid PET/CT (GE Discovery IQ 3Ring; equipped with 16 slice Optima540 CT Model) device. Patients fasted for eight hours before the examination. The images were acquired 60 to 90 minutes after the injection of ^18^F-FDG ((0.14–0.20 mCi/kg) in patients with glucose level less than 200 mg/dL.

### Image Analysis

#### MRI Analysis

DWI interpretation was performed by two radiologists (reader 1, A.V.P.; reader 2, M.O.) independently. The reviewers were blinded to the histopathological diagnosis and other imaging results of the lesions. Each interpreter placed five ellipsoid regions of interest (ROI) boxes on the lesion by referring the solid and most enhancing portion on the contrast-enhanced T1W images. Cystic portions, calcifications and any artifacts were carefully avoided. The mean ADC (ADC_mean_) and minimum ADC (ADC_min_) of each reader’s measurements were stored for final statistical analysis.

#### FDG-PET/CT Analysis

**Figure 2:**
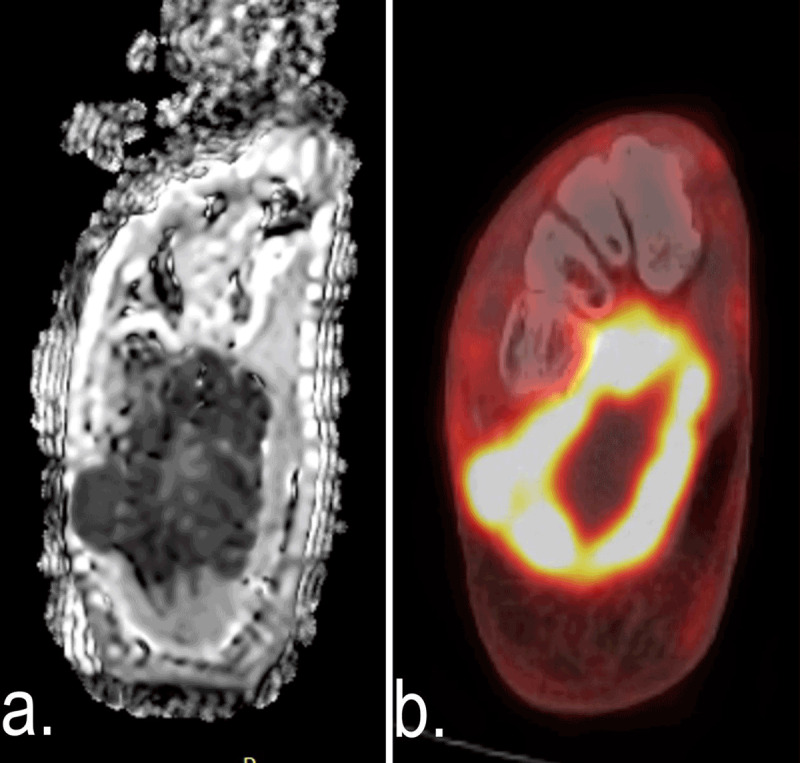
DWI **(a)** and FDG-PET/CT **(b)** images of a 35-year-old male with a soft tissue mass in the right foot. ADC_mean_, ADC_min_ and SUV_max_ of the lesion were 0.55 × 10^–3^mm^2^/s, 0.31 × 10^–3^mm^2^/s and 24.31, respectively. The lesion was diagnosed as undifferentiated pleomorphic sarcoma after core needle biopsy.

**Figure 3:**
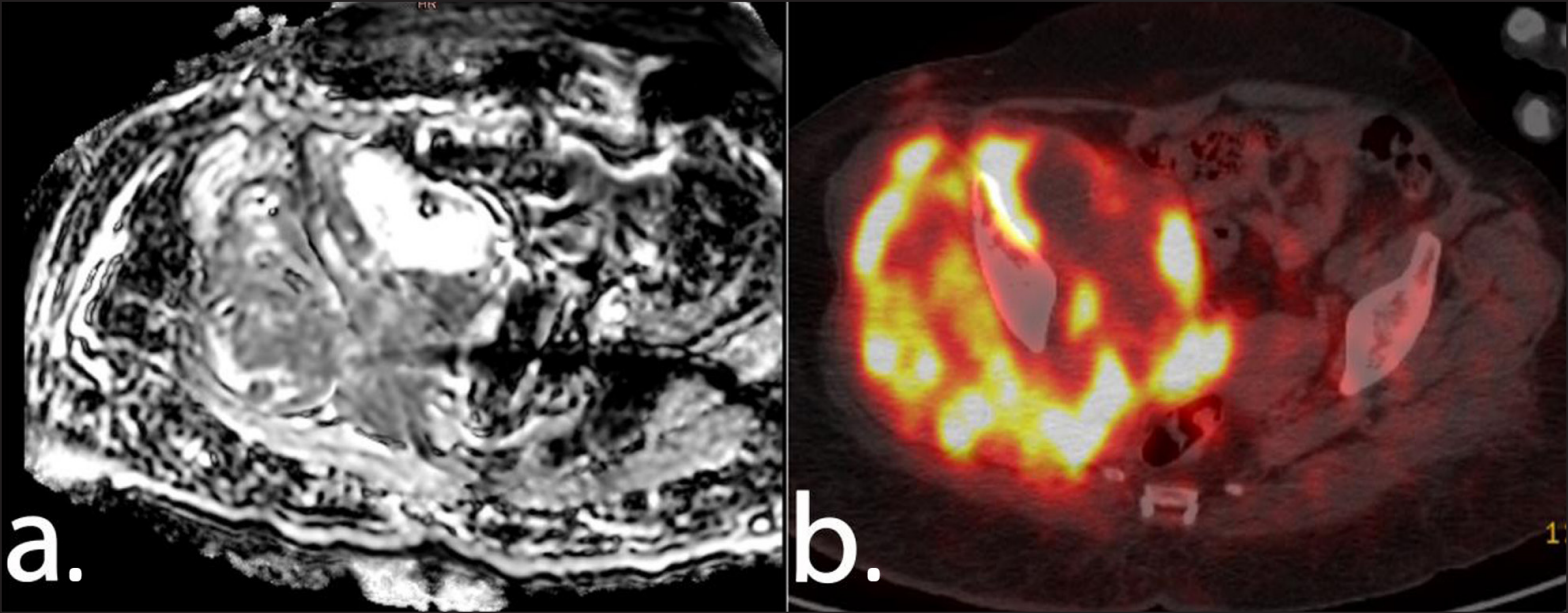
DWI **(a)** and FDG-PET/CT **(b)** images of a 51-year-old female with a soft tissue mass in the right iliac fossa. ADC_mean_, ADC_min_ and SUV_max_ of the lesion were 0.97 × 10^–3^mm^2^/s, 0.60 × 10^–3^mm^2^/s and 13.61, respectively. The lesion was diagnosed as high grade leiomyosarcoma after core needle biopsy.

An experienced nuclear medicine physician (F.C.T.) evaluated the FDG-PET/CT images without knowing the other imaging findings and histopathological results. The lesion standardized uptake value (SUV) was calculated automatically (activity in volume of interest (VOI)/[injected dose*body weight]) using volume-of-interest segmentation of the lesion. The SUV_max_ was defined as the hottest voxel within the VOI (***Figures 2*** and ***3***).

### Histopathological Evaluation

Pathology results of the patients were obtained by reviewing the patients’ chart. Histopathological classification of the soft tissue tumors was according to the 2013 WHO classification. Immunochemical and molecular studies were used to confirm certain diagnosis in certain cases. Tissue specimens were obtained either by surgery (n = 8) or core needle biopsy (n = 17). For four lesions, histopathological diagnosis was not available but the lesions appeared during follow-up imaging so they were accepted as metastases. Core-needle biopsies were performed by an experienced musculoskeletal radiologist (M.B.S.) using a 14–18G automatic core-needle biopsy needle (22 mm excursion; Maxicore, Ankara, Turkey).

### Statistical Analysis

Statistical analysis was performed with the SPSS 15.0 (SPSS Inc.; Chicago, IL, USA). Shapiro-Wilk test was used to assess normal distribution of the continuous data. Data were presented as mean and standard deviation (SD) or median and range for continuous variables, and as frequencies for categorical variables. Student t-test was used to compare the ADC_mean_, ADC_min_ and SUV_max_ between soft tissue and bone tumors. Pearson correlation was used to evaluate the correlation between SUV_max_ and ADC values. The degree of correlation was classified as follows: 0 ≤ r < 2, none; 2 ≤ r < 4, weak; 4 ≤ r < 6, moderate; 6 ≤ r < 8, strong; and 8 ≤ r, very strong correlations. Interobserver agreement of the ADC measurements was assessed using intraclass correlation coefficient. A p value less than 0.05 was indicative of statistical significance.

## Results

Demographic data of the study population is shown in ***Table 1***. A total of 29 lesions in 27 patients were included in the study. There were 18 (62.1%) soft tissue tumors and 11 (37.9%) bone tumors. Their histological subtypes are presented in ***Table 2***. Thirteen lesions (44.8%) were located in the trunk, 13 lesions (44.8%) were located in the lower extremity and three lesions (10.4%) were located in the upper extremity. The mean maximum diameter of the lesions was 9.4 ± 6.2 cm (range: 1.7–25).

**Table 1:** Demographic and clinical characteristics of the study population.


VARIABLE	ALL PATIENTS	PATIENTS WITH STT	PATIENTS WITH BT	P VALUE*

Age (years)	44.85 ± 24.07	45.19 ± 26.16	44.36 ± 21.88	0.932

Gender				0.432

Male	19 (65.5%)	13 (68.4%)	6 (31.6%)	

Female	10 (35.5%)	5 (50%)	5 (50%)	

Maximum diameter (cm)	9.39 ± 6.21	9.78 ± 6.95	8.74 ± 5.01	0.668

ADC_mean_ (x10^–3^mm^2^/s)	0.85 ± 0.28	0.92 ± 0.30	0.75 ± 0.22	0.105

ADC_min_ (x10^–3^mm^2^/s)	0.61 ± 0.27	0.67 ± 0.30	0.50 ± 0.19	0.094

SUV_max_	12.93 ± 9.63	12.23 ± 8.87	14.07 ± 11.11	0.627


*– Derived from the comparison of soft tissue tumors and bone tumors, ADC_mean_– Mean apparent diffusion coefficient, ADC_min_– Minimum apparent diffusion coefficient, BT– Bone tumors, STT– Soft tissue tumors, SUV_max_– Maximum standardized uptake value.

**Table 2:** Histopathological subtypes of the tumors.


TYPES OF TUMORS	NUMBER OF TUMORS

**Soft tissue tumors**	18 (62.1%)

Liposarcoma	3

Rhabdomyosarcoma	1

Undifferentiated spindle cell sarcoma	1

Leiomyosarcoma	3

Angiosarcoma	1

Undifferentiated pleomorphic sarcoma	1

Synovial sarcoma	1

Lymphoma	2

Metastases	5

**Bone tumors**	11 (37.9%)

Ewing sarcoma	2

Chondrosarcoma	1

Plasmocytoma	1

Osteosarcoma	2

Lymphoma	2

Metastases	3


The mean SUV_max_, ADC_mean_ and ADC_min_ of the whole lesions were 12.93 ± 9.63, 0.85 ± 0.28 × 10^–3^mm^2^/s and 0.61 ± 0.27 × 10^–3^mm^2^/s, respectively. There was a weak positive correlation between maximum lesion diameter and SUV_max_ (r = 0.378, p = 0.043). ADC_mean_ and ADC_min_ did not correlate with the maximum diameter of the lesions (r = –0.161, p = 0.403; r = –0.214, p = 0.265 respectively).

**Figure 4:**
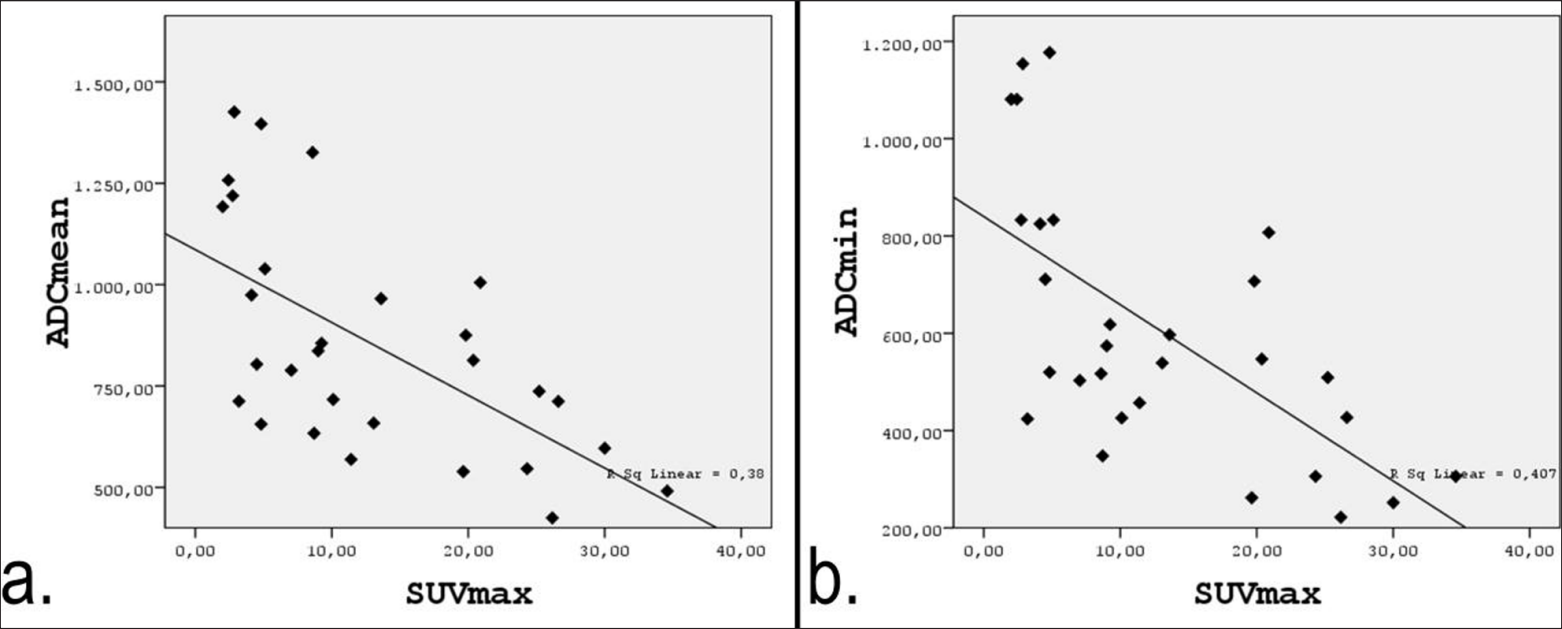
Correlations between the SUV_max_ and both ADC_mean_ **(a)** and ADC_min_ **(b)**.

The relationship between SUV_max_ and ADC values were demonstrated in ***Table 3***. Considering all lesions included in the study, a strong inverse correlation was found between SUV_max_ and ADC_mean_ (r = –0.616, p < 0.001; ***Figure 4***). ADC_min_ showed strong inverse correlation with SUV_max_ (r = –0.638, p < 0.001).

**Table 3:** Correlation analysis results between SUV_max_ and ADC values.


	SUV_MAX_ VS. ADC_MEAN_		SUV_MAX_ VS. ADC_MIN_	

	r	p value	r	p value

**All lesions**	–0.616	<0.001	–0.638	<0.001

**STT**	–0.651	0.003	–0.683	0.002

**BT**	–0.623	0.041	–0.656	0.028


r– correlation coefficient, ADC_mean_– Mean apparent diffusion coefficient, ADC_min_– Minimum apparent diffusion coefficient, BT– Bone tumors, STT– Soft tissue tumors, SUV_max_– Maximum standardized uptake value.

ADC_mean_ and ADC_min_ of the lesions demonstrated a strong inverse correlation with SUV_max_ both separately in soft tissue tumors (r = –0.651, p = 0.003; r = –0.683, p = 0.002) and in bone tumors (r = -0.623, p = 0.041; r = –0.656, p = 0.028).

ADC_mean_ and ADC_min_ measurements of the readers demonstrated almost perfect interobserver agreement (ICC:0.926, CI:0.849–0.965, p < 0.001; ICC:0.883, CI:0.766–0.943, p < 0.001, respectively).

## Discussion

Both DWI and FDG-PET/CT are widely used in clinical practice in the evaluation of musculoskeletal tumors but there are limited studies in the literature comparing them [12,17,18,20,21].

Lee et al. [17] evaluated 25 bone and 32 soft tissue tumors using MRI and FDG-PET/CT. They included both malignant and benign tumors and reported significant inverse correlation between ADC and SUV values. Our study also revealed significant inverse correlation between ADC and SUV, although all of our lesions were malignant. However, their study differed from ours as they did not perform a subgroup correlation analysis of ADC and SUV in bone and soft tissue tumors individually. We found significant correlation between SUV_max_ and ADC values in both soft tissue and bone tumors.

Sagiyama et al. [20] assessed the correlation between ADC and SUV of 35 musculoskeletal tumors using FDG-PET/MRI and reported significant correlation between these two entities. Rakheja et al. [22] evaluated the correlation of ADC and SUV in 52 non-osseous and 17 osseous tumors using FDG-PET/MRI. They reported significant correlation between ADC and SUV values in all osseous and non-osseous lesions; however, subgroup analysis of osseous lesions did not demonstrate correlation between ADC and SUV. Calcification and reparative sclerosis in bone tumors may correspond to low ADC values on DWI. Therefore, low ADC in bone tumors may reflect the calcification of the tissue instead of malignant cells, which might be the underlying reason that ADC_min_ and SUV_max_ did not correlate in bone tumors of their study. In our study, although the difference was not statistically significant, ADC_mean_ and ADC_min_ of bone tumors were lower than those of the soft tissue tumors.

In another study evaluating 136 sarcomas by Rakheja et al. [9], SUV values were found to be correlated with mitotic cell count, presence of necrosis and histological grade of the tumors. Considering the significant heterogeneity and variety of sarcomas, authors suggested performing the biopsy of the lesions from the areas demonstrating maximum SUV uptake for accurate grading and treatment planning. Although we did not assess correlation between SUV, ADC and histological features, correlation between SUV and ADC confirmed that biopsy of musculoskeletal lesions should be performed from the areas with lowest ADC values.

This study has several limitations. First of all, it included only a limited number of tumors. More studies with larger populations are needed to validate our results. Second, this study did not evaluate the correlation between histological parameters and SUV and ADC, as most histological parameters were not available. Third, SUV and ADC measurements were performed by different physicians on different topologies. Therefore, we used blinded comparison of the SUV and ADC measurements. One may hypothesize that a non-blind comparison assessing how often SUV_max_ was on the same area with ADC_nean_ and ADC_min_ may have resulted in more significant correlations. However, comparing these two modalities blindly, settings may be more valuable considering the daily practice. Future studies may assess whether SUV_max_ corresponds to the same area with ADC_min_ and vice versa.

## Conclusion

In conclusion, SUV_max_ of FDG-PET/CT has a strong but inverse correlation with the ADC_mean_ and ADC_min_ values obtained from DWI in musculoskeletal tumors.
